# Response of a clinical Escherichia coli strain to repeated cefquinome exposure in a piglet tissue-cage model

**DOI:** 10.1186/s12917-015-0486-6

**Published:** 2015-07-25

**Authors:** Mengxiao Gu, Nan Zhang, Longfei Zhang, Mingpeng Xiong, Yuanyuan Yang, Xiaoyan Gu, Xiangguang Shen, Huanzhong Ding

**Affiliations:** National Reference Laboratory of Veterinary Drug Residues (SCAU), College of Veterinary Medicine, South China Agricultural University, Guangzhou, 510642 China

**Keywords:** Tissue-cage model, *in vivo* PK/PD, Cefquinome, A clinical *Escherichia coli* strain, Piglets

## Abstract

**Background:**

In order to provide some basis for effective dosage regimens that optimize efficacy with respect to bacteriological and clinical cures, the *in vivo* activity of cefquinome against a clinical *Escherichia coli* (*E.coli*) strain (the minimum inhibitory concentration value for this strain equals to the MIC_90_ value of 0.25 μg/ml for 210 *E.coli* strains isolated from pigs) was investigated by using a piglet tissue-cage infection model. The aim was to elucidate the pharmacokinetic/pharmacodynamics (PK/PD) index associated with cefquinome efficacy, and then to identify the magnitude of the PK/PD parameter required for different degree of efficacy in clinical treatment.

**Results:**

Tissue-cage infection model was established in piglets, and then the animals received intramuscular injection of cefquinome twice a day for 3 days to create a range of different drug exposures. The tissue-cage fluid was collected at 1, 3, 6, 9 and 12 h after every drug administration for drug concentration determination and bacteria counting. Different cefquinome regimens produced different percentages of time during that drug concentrations exceeded the MIC (%T > MIC), ranging from 0 % to 100 %. Cefquinome administration at 0.2, 0.4, 0.6, 0.8, 1, 2 and 4 mg/kg reduced the bacterial count (log_10_ CFU/mL) in tissue-cage fluid by −1.00 ± 0.32, −1.83 ± 0.08, −2.33 ± 0.04, −2.96 ± 0.16, −2.99 ± 0.16, −2.93 ± 0.11, −3.43 ± 0.18, respectively. The correlation coefficient of the PK/PD index with antibacterial effect of the drug was 0.90 for %T > MIC, 0.62 for AUC_0–12_/MIC, and 0.61 for C_max_/MIC, suggesting the most important PK/PD parameter was %T > MIC. A inhibitory form of sigmoid maximum effect (E_max_) model was used to estimate %T > MIC, and the respective values required for continuous 1/6-log drop, 1/3-log drop and 1/2-log drop of the clinical *E.coli* count during each 12 h treatment period were 3.97 %, 17.08 % and 52.68 %.

**Conclusions:**

The data derived from this study showed that cefquinome exhibited time-dependent killing profile. And from the results of the present study, it can be assumed that when %T > MIC reached 52.68 %, cefquinome could be expected to be effective against a clinical *E.coli* strain for which the MIC value is below 0.128 μg/ml (3-log drop of bacteria count can be achieved after six successive administrations for 3 days).

## Background

Overuse and misuse of antimicrobial drugs have favoured the growth of resistant organisms. Resistance can spread to other microbial populations (including those bacteria not previously exposed to antimicrobial agents), jeopardizing humans and animals. Among the documented misuses contributing to drug resistance are inappropriate dosage regimens (dose, dosage interval, duration of treatment, route and conditions of administration) (Anonymous 1998). Rational antibiotic therapy requires dosage regimens to be optimized, not only to guarantee clinical efficacy, but also to minimize the selection and spread of resistant pathogens [1].

Jacobs thought pharmacokinetics (PK) and pharmacodynamics (PD) should be considered to establish optimal dosing regimens for antimicrobials [2]. Besides, Toutain and Lees thought that the parameters derived from PK/PD modelling may be used as an alternative and preferred approach to dose titration studies for selecting rational dosage regimens (both dose and dosing interval) for further evaluation in clinical trials [3].

Since 1935 when Domagk demonstrated that prontosil provided effective therapy in a murine pneumococcal infection model, the use of animal infection models has become an integral part of the evaluation of new antimicrobial agents and new therapeutic approaches for clinical trials in humans. Compared with *in vitro* studies, animal models provide a dynamic interaction of multiple factors relating to the host, drug and pathogens [4]. Tissue cages were first used by Guyton [5] to study interstitial fluid physiology and composition. In the veterinary field, the tissue-cage model has been used to study the PK/PD integration of antimicrobial drugs in several species, such as sheep [6], goats [7], pigs [8, 9] and calves [10, 11].

Cefquinome is a fourth generation broad-spectrum cephalosporin developed solely for veterinary use and approved for the treatment of respiratory tract disease, acute mastitis and foot rot in cattle, calf septicemia, metritis-mastitis-agalactia syndrome in sows and respiratory diseases in pigs [12, 13]. It also has been used to prevent severe pneumonia in piglets (caused by pathogens such as *Actinobacillus pleuropneumonia*, *Klebsiella pneumonia*, and *Streptococcus pneumonia*) and to establish pig herds free of pneumonia by combining its use with strategic vaccination [14].

Although PK and *ex vivo* PD of cefquinome against *Escherichia coli* (*E.coli*) 25922 has been explored previously [9], there is no report so far concerning the *in vivo* PK/PD analysis of cefquinome in piglets. Previous studies about *in vivo* PK/PD of the antibacterial agent were mostly against various standard strains, hardly any against the clinical bacterial strains. Therefore, in the present study, we collected 210 *E.coli* clinical strains isolated from pigs with common colibacillosis, and one strain whose minimum inhibitory concentration (MIC) equals to the MIC_90_ of 210 strains was used for tissue-cage infection model. After the tissue-cage infection model was established, the animals were treated with multiple dosing, which was consistent with the actual situation of clinical treatment. The aim of this study was to characterize the PK/PD parameter that is predictive of the efficacy of cefquinome against the clinical *E.coli* strain in a piglet tissue-cage infection model for the first time. Furthermore, magnitude of the PK/PD parameter will also be determined for bactericidal efficacy in clinical treatment. It is proposed that these findings can provide a rational approach to designing dosage schedules that optimize efficacy with respect to bacteriological and clinical cures.

## Methods

### The selection of the test strain

In this study, 210 *E.coli* strains isolated from pigs with common colibacillosis were used to determine the MIC_90_ value. The MIC values were determined by an agar dilution method as a preliminary screening, according to Clinical and Laboratory Standards Institute (CLSI) reference methods [15]. Strains whose MIC values equal to the MIC_90_ value of 210 strains were selected for pathogenicity test.

Pathogenicity test (the license number: SCXK 2011–0015) was carried out with 30 Kunming mice (SPF grade, specific pathogen free grade) purchased from animal experimental center of southern medical university. Every three mice were infected by intraperitoneal injection of 0.2 mL *E.coli* saline suspension (1 × 10^9^ colony-forming unit, CFU/mL). The blank control group was given the same amount of saline. The mice were observed every 3 h after the injection until 72 h. If there were two or three mice dead in a group, then the inoculant given to this group was considered to be of high pathogenicity. The dead mice were dissected, and gram staining, microscopy and biochemical identification were carried out for the bacteria isolated from liver to determine whether the acquisition was the same as the inoculant.

The bacteria strain that was considered to be of high pathogenicity was selected out. In the present study, the bacterium was inoculated into the tissue-cage. Since cefquinome acts on the bacterium in the tissue-cage fluid, we need to measure the MIC of cefquinome against the selected strain in the tissue-cage fluid. Due to the tissue-cage fluid was limited, we determined the MIC once again by a micro dilution assay, and the matrix was the tissue-cage fluid.

The target strain was sent to Guangzhou King med Center for Clinical Laboratory Co., Ltd. for further bacterial identification, and the detection method was GB/T4789.6-2003.

### Antimicrobials, chemicals

Cefquinome Sulfate Injection (Cobactan, the Batch Number A621B01) was purchased from Merck&Co. INC. Cefquinome standard was purchased from China Institute of Veterinary Drugs Control, (Beijing, P.R. China). Pentobarbital sodium was from Jian Yang Biotechnology Co., Ltd. Procainamide hydrochloride was purchased from Xin zheng Co., Ltd., Tianjin Pharmaceutical Group.

### Animals and surgical procedures

Sixteen healthy castrated cross-bred piglets (Duroc × Landrace × Yorkshire) were used, weighing 25-30 kg. They were housed in a ventilated barn individually. Each animal was fed antibiotic-free food (guangchubao premix feed for pig, Guangzhou Zhongwang Feed Company) twice a day and water was available *ad libitum*. The tissue cages were prepared from medical grade silicone rubber tubing and modified slightly from similar cages described by Sidhu [16]. The length of the tissue cage was 65 mm, the internal and external diameter were 13 mm and 18 mm, respectively. Each cage had 24 identical holes and the surface area of each hole was 9.6 mm^2^, therefore the total exchange surface was 2.3 cm^2^. Two sterile tissue cages were implanted subcutaneously in each animal, one on each side of the neck approximately equidistant from the jugular vein and spinal cord. Surgical insertion was carried out under deep general anesthesia induced with pentobarbital sodium and local infiltration anesthesia by the injection of procainamide hydrochloride. Animals were allowed to recover from surgery for 3–4 weeks to permit wound healing and the growth of granulation tissue into and around the cages. After the surgery, piglets were treated with intramuscular penicillin twice a day for 3 days to prevent infection. The compound aminopyrine injection was administrated for post-operative analgesia simultaneously. About 3–4 weeks after implantation, the surface of the cage becomes encapsulated by connective tissue, and the interior is filled with tissue-cage fluid. All procedures involving animals were conducted under the close supervision and guidance of an experienced veterinary surgeon. The experimental protocol was approved by the Committee on the Ethics of animals of South China Agricultural University (Approval number 2014–04; 10 February 2014).

### Animal infection and treatment

Before the infection experiment, 0.5 mL tissue-cage fluid was sampled from the tissue cage to confirm the tissue-cage fluid was sterile. The animal with bacterial present in the tissue-cage fluid before inoculation of *E.coli* was excluded from the study. In the current study, no piglets were excluded from this study due to a prior infection within the tissue cage. The tissue cages were inoculated with 1 mL of *E.coli* saline suspension (1.4 × 10^8^ CFU/mL). The piglets remained infected without treatment for two days.

Animals were divided randomly into 8 groups (2 piglets, 4 tissue cages/group). Cefquinome sulfate injection was administrated intramuscularly at different dosage for different group after bacterial infection. Seven doses (0.2, 0.4, 0.6, 0.8, 1, 2, 4 mg/kg) of cefquinome were given (twice a day for 3 days) to create a range of different drug exposures. The control group received sterile physiological saline (1 mL) simultaneously in the same way.

### Bacteriological examination of tissue-cage fluid

A 0.5 mL sample of tissue-cage fluid was removed from the tissue cage prior to every drug administration during the whole treatment and 12 h, 24 h and 36 h after the last treatment. Within 1 h after sampling, 100 μL of the tissue-cage fluid was serially 10-fold diluted in sterile physiological saline. From each dilution, 0.1 mL was plated onto MacConkey Agar Plate for overnight incubation at 37 °C for manual colony counts. The theoretical limit of detection of this procedure was 20 CFU/mL. The efficacy of cefquinome was calculated as the decrease in the amount of the bacteria over 3 days of treatment compared with the bacteria levels before the treatment.

The emergence of resistance under the dosing regimens in this study was assessed by measuring the MIC for *E.coli* recovered from tissue-cage fluid over the 3-day treatment period and 24 h posttreatment observation period. The MIC of cefquinome for the test strain was determined in duplicate using the micro dilution technique established by the CLSI [15].

### Pharmacokinetic sampling and drug analysis

The tissue-cage fluid sample (0.5 mL) was collected from the inserted tissue cages with a syringe at 1, 3, 6, 9 and 12 h after each dosing. Samples were clarified by centrifugation at 3000 g for 10 min and stored at −20 °C until analyzed.

Cefquinome concentrations in tissue-cage fluid were analyzed by an Agilent 1200 series high performance liquid chromatography and an Agilent 6400 triple quadrupole mass spectrometer equipped with an electrospray ionization source (high performance liquid chromatography tandem mass spectrometry, HPLC-MS/MS, Agilent Technologies, and USA). The chromatographic separation was achieved on a Phenonenex BDS C_18_ column (150 mm × 2 mm; internal diameter, 5 μm, Phenomenex Technologies) at 40 °C with a thermostat column oven (Agilent 1200 series, Agilent Technologies). The mobile phase consisted of solution A (water with 0.1 % formic acid, V/V) and solution B (acetonitrile) at 0.25 mL/min flow rate. The gradient elution was: 0–1 min, 5 % B; 1–5.5 min, 60 % B; 5.5-10 min, 5 % B. The injection volume was 5 μL. All tissue-cage fluid samples were allowed to thaw at room temperature prior to analysis and then aliquots 200 μL tissue-cage fluid were added to a 1.5 mL micro centrifuge tube. Protein precipitation was accomplished by adding the same volume acetonitrile to the samples. After vorexing for 30s, and centrifuging at 3000 g for 10 min. 200 μL clear supernatant were pipetted into a fresh vial and 800 μL of water were added. After vortex-mixing for 15 s, the samples were filtered through a 0.22 μm nylon syringe filter (JinTeng Experiment Equipment Company) and then injected into an auto sampler vial. Cefquinome quantification in the tissue-cage fluid was linear within a range of 25–5000 ng/mL and the correlation coefficieent was >0.99. The lower limit of quantitation was 5 ng/mL. The recoveries of cefquinome in tissue-cage fluid were 90.2 ± 3.17 % (mean ± standard deviation, *n* = 5). The coefficients of variability (CV %) were all < 8 % for both intra-assay and inter-assay variation.

### Pharmacokinetics/pharmacodynamics (PK/PD) integration and modeling

The PK/PD parameters, i.e., %T > MIC, AUC_0–12_/MIC (AUC, area under the curve) and C_max_/MIC (C_max_, the maximum concentration) were calculated for each tissue cage, from 0 to 12 h after every drug administration. This is due to the dosage regimens. In this study, we treated the animals with continuous multiple dosing (twice a day for 3 days), with the dosing interval of 12 h. %T > MIC (the percentage of time that drug concentration remains above the MIC) and C_max_ (the maximum concentration measured in the tissue-cage fluid) were taken directly from the concentration-time profiles. AUC_0–12_, the area under the concentration-time curve, was calculated by the trapezoidal rule. The dose–response effect of the drug was analyzed by fitting the %T > MIC, AUC_0–12_/MIC and C_max_/MIC ratio versus the reduction of bacteria count with a inhibitory form of the sigmoid E_max_ model, through which we can further elucidate the PK/PD indices required for various degrees of antibacterial efficacy. The equation used to characterize the PK/PD parameters and log_10_CFU/mL could be described as the following:

$$ E={E}_{max}-\frac{\left({E}_{max}-{E}_0\right)\times {C}_e^N}{C_e^N+E{C}_{50}^N} $$

Where *E* is the antibacterial effect, defined as the change of bacterial count (log_10_ CFU/mL) after every drug administration (during 12 h treatment period); *E*_*max*_ is the change of bacterial count in control sample (absence of cefquinome) after 12 h incubation; *E*_*0*_ is the maximum antibacterial effect, determined as the maximum change of bacterial count every 12 h; *C*_*e*_ is the PK/PD parameter being examined (e.g., %T > MIC, AUC_0–12_/MIC, or C_max_/MIC); *EC*_*50*_ is the value of PK/PD index of drug producing 50 % of the maximum antibacterial effect, and N is the Hill coefficient that describes the steepness of the effect curve. These PD indices were calculated by running the WinNonlin (Pharsight Corporation, Mountain View, CA, USA) sigmoid *E*_max_ model to fit the experimental data collected. Nonlinear regression analysis was used to determine which PK/PD index best correlated with the bacterial reduction. Spearman’s rank correlation coefficient was calculated to evaluate the relationship between drug efficacy and the PK/PD indices.

## Results

### MICs of cefquinome for 210 clinical *E.coli* strains

The MICs of cefquinome for 210 clinical *E.coli* strains were shown in Table 1. From the above results, it can be determined that the MIC_90_ value was 0.25 μg/mL. So there were nine strains of bacteria meeting the requirements. In the pathogenicity test, bacteria derived from the dead mice were confirmed to be the same as the inoculant. One strain with highly pathogenicity was selected from nine inoculations. The MIC value of the strain in tissue-cage fluid was further measured to be 0.128 μg/mL. The Serotype of the strain was identified by Guangzhou King med Center for Clinical Laboratory Co., Ltd. The bacteriology type was EIEC (Enter invasive *Escherichia Coli*) and the serotype was O29 (report number was HT14020480-1).

**Table 1:** The MICs of cefquinome for 210 clinical *E.coli* strains

MIC(μg/mL)	Number of strains	Cumulative strains
0.0325	53	53
0.0625	102	155
0.125	30	185
0.25	9	194 (>189)
0.5	4	198
1	3	201
2	6	207
4	3	210

The MIC values were determined by an agar dilution method. From the above results, it can be determined that the MIC_90_ value was 0.25 μg/ml

### The pharmacokinetics of cefquinome

The mean values of AUC_0–12_ and C_max_ of cefquinome in the tissue-cage fluid following multiple dosing at 0.2, 0.4, 0.6, 0.8, 1, 2 and 4 mg/kg of body weight are shown in Table 2. The time course of cefquinome concentrations in the tissue-cage fluid was analyzed using the noncompartmental method. The AUC_0–12_ and C_max_ in the tissue-cage fluid increased in a nonlinear fashion with dose escalation. The AUCs from 0 to 12 h after every administration, as determined by trapezoidal rule, ranged from 1.346 ± 0.046 to 20.73 ± 0.064 μg · h/L. The peak concentrations were directly observed from the concentration-time curve, ranging from 0.135 ± 0.004 to 2.069 ± 0.070 μg/mL.

**Table 2:** The pharmacokinetics parameters of cefquinome following multiple-dose in the *E.coli*-infected piglets

Cefquinome dose	AUC_0–12_	C_max_
(mg/kg of body weight)	(μg · h/mL)	(μg/mL)
0.2	1.346 ± 0.046	0.135 ± 0.004
0.4	1.553 ± 0.066	0.170 ± 0.007
0.6	2.802 ± 0.044	0.286 ± 0.001
0.8	3.693 ± 0.088	0.396 ± 0.008
1	4.230 ± 0.013	0.448 ± 0.003
2	11.157 ± 0.048	1.175 ± 0.024
4	20.730 ± 0.0640	2.069 ± 0.070

The AUC_0–12_ and C_max_ were the mean values of multiple-dose (six times). AUC_0–12_, area under the concentration-time curve from 0 to 12 h after every administration, was determined by use of the trapezoidal rule; the C_max_ was directly from the concentration-time curve of every administration. The values shown are means ± standard deviations

### PK and PD parameter determination

**Fig. 1:**
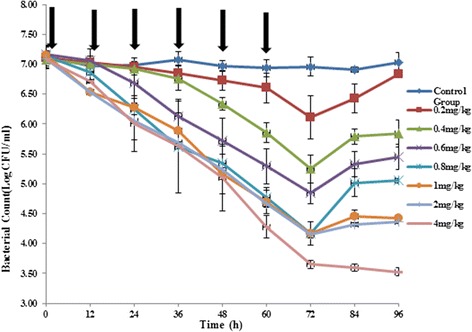
The antibacterial time-kill curve of cefquinome in the tissue-cage model. Two days after infection, seven doses (0.2, 0.4, 0.6, 0.8, 1, 2, 4 mg/kg) of cefquinome were given twice a day for 3 days (indicated by the arrow). The bacterial count in the tissue-cage fluid was monitored every 12 h, prior to every drug administration during the whole treatment and 12 h, 24 h and 36 h after the last treatment. The values are means ± standard deviations (two piglets, four tissue-cages)

The efficacy of cefquinome was assessed by determining the number of viable bacteria in the tissue-cage fluid over 3-day dosing period. The antibacterial time-kill curves are shown in Fig. 1. Cefquinome administrated at 0.2, 0.4, 0.6, 0.8, 1, 2 and 4 mg/kg reduced the bacterial count (log_10_ CFU/mL) in tissue-cage fluid by −1.00 ± 0.32, −1.83 ± 0.08, −2.33 ± 0.04, −2.96 ± 0.16, −2.99 ± 0.16, −2.93 ± 0.11, −3.43 ± 0.18, respectively. In detail, the reduction of bacterial count after every drug administration (at 12 h intervals) is shown in Table 3. The bacterial density in the tissue-cage fluid of untreated controls approximately remained at 10^7^ CFU/ml during the whole course of treatment. Besides, for the *E.coli* recovered from the tissue-cage fluid over the 3-day treatment and 24 h posttreatment observation period, the MIC value did not change, that is to say, the bacterial strain remained susceptible to cefquinome throughout the study period.

**Table 3:** The reduction of bacteria count for each every drug administration at seven dosages

Times of drug administration	Doses (mg/kg of body weight)						
	0.2	0.4	0.6	0.8	1	2	4
1st	−0.08	−0.08	−0.10	−0.26	−0.62	−0.56	−0.36
2nd	−0.08	−0.07	−0.38	−0.62	−0.26	−0.48	−0.71
3rd	−0.10	−0.16	−0.57	−0.61	−0.39	−0.36	−0.38
4th	−0.12	−0.43	−0.40	−0.26	−0.72	−0.45	−0.53
5th	−0.12	−0.48	−0.43	−0.57	−0.48	−0.57	−0.82
6th	−0.50	−0.61	−0.45	−0.61	−0.52	−0.51	−0.63
Total	−1.00	−1.83	−2.33	−2.96	−2.99	−2.93	−3.43

The reduction of bacterial count after every drug administration (at 12 h dosing intervals) is expressed in negative value. And the last line is the total reduction of bacterial count after six times of drug administration

**Fig. 2:**
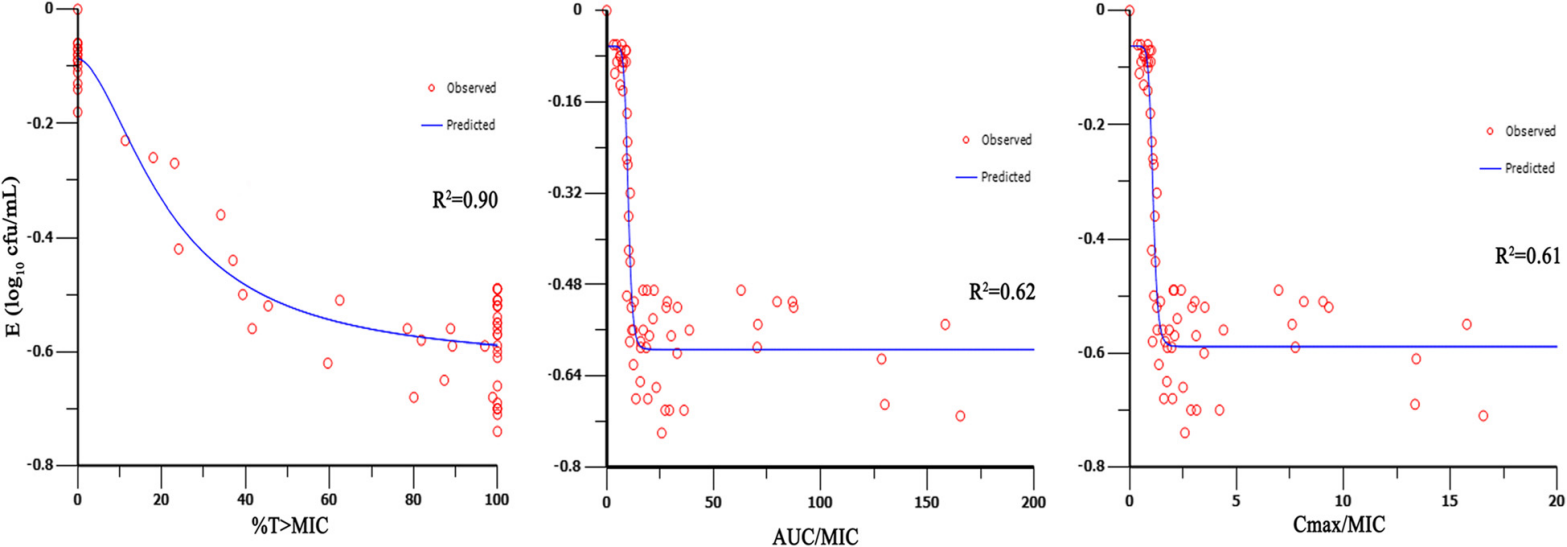
Relationships between %T > MIC, AUC_0–12_/MIC, C_max_/MIC and E. E is defined as the reduction of bacterial count after every drug administration, at 12 h dosing intervals. The lines represent the best model fits of the data. R^2^ is the correlation coefficient

The relationships between antibacterial effect and PK/PD parameters (%T > MIC, AUC_0–12_/MIC, and C_max_/MIC) are shown in Fig. 2. The %T > MIC was the parameter that most strongly correlated with efficacy (R^2^ = 0.90), whereas the relationship between efficacy and other parameters was not nearly as strong (for AUC_0–12_/MIC, R^2^ = 0.62; for C_max_/MIC, R^2^ = 0.61). In this study with a clinical *E.coli* strain, even the highest dose levels did not achieve a virtual eradication effect (4-log drop of bacterial count) over 3-day treatment period. The above studies indicated that treatment efficacy depended on the percentage of time that drug concentration remained above the MIC.

### Magnitudes of the PK/PD parameter required for efficacy

The relationships between antibacterial efficacy and %T > MIC were assessed by using the inhibitory sigmoid *E*_max_ model, and the obtained parameters of *E*_0_, *E*_max_, the Hill coefficient and %T > MIC values required for various degrees of antibacterial efficacy are presented in Table 4. The estimated %T > MIC values for continuous 1/6-log drop, 1/3-log drop and 1/2-log drop of bacterial count were 3.97 %, 17.08 % and 52.68 % during 12 h treatment period of cefquinome, respectively.

**Table 4:** The values of PK/PD parameter and %T > MIC required to achieve various degrees of antibacterial efficacy

Parameters	Values
*E*_*max*_ (△log CFU/mL)	0.09
*E*_*0*_ (△log CFU/mL)	−0.54
*E*_*max*_ - *E*_*0*_ (△log CFU/mL)	0.63
%T > MIC for 1/6-log drop (%)	3.97
%T > MIC for 1/3-log drop (%)	17.08
%T > MIC for 1/2-log drop (%)	52.68
Slope (*N*)	1.73

*E*_max_, the difference of the bacterial count in the control sample (absence of cefquinome); *E*_0_, the maximum antibacterial effect during 12 h treatment period; *N*, the Hill coefficient that describes the steepness of the effect curve. %T > MIC, the values of cefquinome required to achieve 1/6-log drop, 1/3-log drop and 1/2-log drop against the clinical *E.coli* strain during 12 h treatment period

## Discussion

Antimicrobial PK/PD analyses to identify the PD activity of antimicrobial agents through the integration of the PK properties, *in vitro* potency (MIC), and outcome is one approach that has proven helpful to the design of effective dosing regimens in humans and animals [4, 17].

Many reports concerning PK/PD studies of antimicrobials against various species of bacteria have been reported, including voriconazole against *Candida albicans* [18], biapenem against *Psudomonas aeruginosa* [19], ceftaroline fosamil against Methicillin-Resistant *Staphylococcus aureus* [20], RWJ-54428 against *Staphylococcus aureus*, *Streptococcus pneumoniae*, and *Enterococcus faecalis* [21], and piperacillin against *E.coli* [22], cefquinome against *Staphylococcus aureus* [23].

It has been reported that bacteria have different growth behavior *in vitro* and *in vivo*, this event being particularly associated to the medium conditions and the source of substrates. In an *in vitro* environment, bacteria differ from those cultivated *in vivo* in the area of amino acid composition, synthesis of toxic metabolites and metabolic rate [22]. The bacteria can adhere to cells and fibers present in the matrix, making the access of the drug more difficult in the *in vivo* infection model. But *in vivo* infection model really reflects the interaction between the body, drug and pathogens. Hence, in the current study, we describe the PK/PD profile of cefquinome against a clinical *E.coli* strain in a more realistic scenario by using an *in vivo* experimental infection model.

In the preliminary study, we treated the infected animals with a single dose of 0.5, 1 and 2 mg/kg b.w. However, the results were unsatisfactory. After the drug administration, the bacteria counts in the tissue-cage fluid have certain reduction (all less than 1-log for 0.5, 1 and 2 mg/kg) in the first 12 h. But the bacteria count increased again during 12-24 h. The duration of time that the drug inhibited the growth of the bacteria was short. By monitoring the drug concentration, we discovered that with the course of drug elimination, the drug concentration exceeded the MIC momently and then fell below the MIC. Then we treated the infected animals with multiple dosing and reduced the dosing interval to make the T > MIC longer. The experiment results indicated that the values of %T > MIC rose gradually with increasing dosage, varying from 0 % to 100 %.

The previous studies about *in vivo* PK/PD of the antibacterial agent were mostly against various standard strains, hardly any against a clinical bacterial strain. In this study, we measured the MIC_90_ of cefquinome for 210 *E.coli* strains isolated from pigs, and investigated the response of a clinical *E.coli* strain (the MIC value for this bacteria strain equals to the MIC_90_ value) to repeated cefquinome exposure in an *in vivo* experimental infection model. This is critical for optimal dosage regimens to treat the diseases caused by *E.coli.*

The results of the current multiple-dosing regimen studies confirmed that %T > MIC has a favorable correlation with the efficacy of cefquinome. The results obtained in the present study were consistent with the results of other β-lactams in the previous studies. For instance, Andes [24] reported that the *in vitro* bactericidal effect was time-dependent and that %T > MIC was an important PK/PD parameter for β-lactams with a weak PAE. There were also studies [25] reporting that cephalosporins act as time-dependent antimicrobials, and the most appropriate PK/PD parameter to describe drug efficacy was the time that the drug concentration exceeds the MIC. As to cefquinome, it has been reported that %T > MIC was the PK/PD index that best described the efficacy of the antibacterial agent in a neutropenic mouse thigh model of *Staphylococcus aureus* infection [23].

Many *in vitro* and *in vivo* PK/PD models have suggested that the magnitude of the %T > MIC is predictive index of cephalosporin efficacy. In those reports, %T > MIC ranges from 25 % to 70 % for defined therapeutic endpoint [26–28]. The previous studies about Ceftaroline fosamil (PPI-0903 or TAK-599) against *E.coli* 25922 [20] showed that the bacteriostatic effect, a 1-log drop and a 2-log drop were achieved when the %T > MIC reached 32 %, 44 % and 50 %, respectively. With respect to RWJ-54428 against *Enterococcus faecalis* in the murine neutropenic thigh infection model, it was reported [21] that the ranges of %T > MIC for bacteriostatic and maximal effect (the maximal reduction in bacterial count was 1.7) were 30 % to 46 % and 55 % to 60 %, respectively. The study of cefquinome in a neutropenic mouse thigh model of *Staphylococcus aureus* infection indicated that the %T > MICs ranged from 30.28 to 36.84 % for bacteriostatic effect, 34.38 to 46.70 % for a 0.5-log drop, and 43.50 to 54.01 % for a 1-log drop [23].

In the current study, an analysis of the results showed that during 12 h treatment period, continuous bacterial count reduction by 1/6-log, 1/3-log and 1/2-log drop were achieved when the %T > MIC reached 3.97 %, 17.08 % and 52.68 %, respectively. These values are not identical to the activities of other cephalosporin. This may be due to different time span (12 h in this study versus 24 h in other studies), or diversity of animal models, different bacteria species or inoculation level in these studies.

## Conclusions

The current study characterized the *in vivo* response of a clinical *E.coli* strain to cefquinome in a piglet tissue-cage infection model after different dosing. The data derived from this study showed that cefquinome exhibit time-dependent killing against *E.coli* in an *in vivo* model, and the most important PK/PD parameter was %T > MIC. From the results of the present study, it can be assumed that when %T > MIC reached 52.68 %, cefquinome could be expected to be effective against bacterial strains for which the MIC value is below the MIC_90_ of 0.128 μg/ml (3-log drop of bacterial count can be achieved after six times of drug administration, twice a day, 3 days). Since the current study investigated the *in vivo* PK and PD of cefquinome against a clinical *E.coli* strain which represents the MIC_90_ for 210 clinical strains, thus the results are meaningful for designing reasonable clinical treatment regimens.

## Abbreviations

E.coli: Escherichia coli
MIC: Minimum inhibitory concentration
PK: Pharmacokinetics
PD: Pharmacodynamics
AUC: Area under the curve
C_max_: The maximum concentration
CFU: Colony forming unit
HPLC-MS/MS: High performance liquid chromatography tandem mass spectrometry
CV: Coefficients of variability
CLSI: Clinical and laboratory standards institute
SPF: Specific pathogen free
EIEC: Enter invasive Escherichia Coli
